# The Hydraulic Driving Mechanisms of Cyanobacteria Accumulation and the Effects of Flow Pattern on Ecological Restoration in Lake Dianchi Caohai

**DOI:** 10.3390/ijerph16030361

**Published:** 2019-01-28

**Authors:** Peng Zhang, Rui-Feng Liang, Peng-Xiao Zhao, Qing-Yuan Liu, Yong Li, Kai-Li Wang, Ke-Feng Li, Ying Liu, Peng Wang

**Affiliations:** 1 State Key Laboratory of Hydraulics and Mountain River Engineering, Sichuan University, Chengdu 610065, China; sgz_scu@163.com (P.Z.); qingyoan.l1u@gmail.com (Q.-Y.L.); li_yong@scu.edu.cn (Y.L.); wangkaili_scu@163.com (K.-L.W.); kefengli@scu.edu.cn (K.-F.L.); 2 Power China Hua Dong Engineering Corporation Limited, Hangzhou 310014, China; zpx199207@163.com; 3 Power China Kunming Engineering Corporation Limited, Kunming 650051, China; Environment_Y@163.com (Y.L.); w133505@126.com (P.W.)

**Keywords:** wind-driven current, inflow/outflow, water quality response, ecological restoration, ecological operation, Lake Dianchi Caohai

## Abstract

Due to rapid increases in socioeconomic development and the human population over the past few decades, the shallow lakes in China have suffered from eutrophication and poor water quality. The conditions in Lake Dianchi Caohai, which is in the northern part of Lake Dianchi, are considered the most serious. The ecological restoration of Lake Dianchi Caohai began in the late 1980s. Lake managers and the public have been puzzled by the lack of a significant response of the water quality to the flow pattern despite the tremendous investment in water quality improvements. Therefore, lake managers desperately need to understand the responses of pollutant behaviors to proposed management measures. In this paper, a depth-averaged two-dimensional hydrodynamic and water quality model based on hydrological data, measured lake bed elevation, and water quality data is developed to simulate the flow field and water quality of Lake Dianchi Caohai. This model was validated using water quality data from the Caohaizhongxin site in 2016, and a close agreement was found between the model results and observations. Wind-driven circulation in Lake Dianchi Caohai was observed in the model results, which revealed that the lake flow pattern was dominated by wind-driven circulation, while the inflow/outflow played only a subsidiary role during this period. The formation of the wind-driven current in Lake Dianchi Caohai could be roughly divided into three stages. The hydrodynamic processes connected with the distribution of chlorophyll a are evaluated and discussed to adequately understand the hydraulic mechanisms driving the accumulation of cyanobacteria. Moreover, we designed three scenarios after comparing all possible operation scenarios to analyze the contributions of each different operation scenario to the water quality improvements. The optimal ecological operation scenario which has the best impacts on the water quality, especially the reduction in Chla and NH_3_-N concentration, is proposed based on our comprehensive analysis. The water quality improvement and management suggestions proposed in this paper are based on lake flow patterns and make up for previous studies that did not consider the effects of hydraulic characteristics on water quality improvement in Lake Dianchi Caohai.

## 1. Introduction

Shallow lakes, which provide nutrients and habitats for aquatic animals and freshwater and food for humans, are distributed in densely populated areas with low-lying terrain in China [1,2,3,4]. Substantial changes to ecosystem functions are occurring in many shallow lakes, such as the decrease in water area, the degeneration of aquatic ecology, the proliferation of cyanobacterial blooms, and the reduction in drinking water supplies due to severe water quality problems, which have all occurred in Dianchi, Taihu, and Chaohu lakes [5,6,7,8,9]. There are strong relationships among the water quality of lakes, human activities, terrestrial environments, and climate environments [9,10,11]. The water quality is directly negatively affected by point source pollution through severely polluted inflow branches and nonpoint source pollution from intensive agricultural activities, sewage discharge, and urban runoff [4,12]. The water quality and eutrophication status of a lake will deteriorate when the pollution loading into the lake exceeds the carrying capacity of the water environment [12,13]. Moreover, climate changes accompanied by rising air temperatures and changes in wind speed and direction can indirectly affect the water temperature structures of lakes and change the hydrodynamic conditions in shallow lakes to accelerate the occurrence of water pollution and toxic cyanobacterial blooms, which are the key characteristics of eutrophication [3,10,14,15]. All water quality problems will degrade the stability of lake ecosystems and landscape values, reduce water functions, and lead to the reduction in available water resources, even causing irreversible harm to human health [16,17,18,19]. In recent decades, the conservation of the water quality of shallow lakes has become a major concern for society, and improving water quality is urgently needed for heavily polluted shallow lakes [16,20,21].

Ecological restoration is recognized as an effective way to mitigate eutrophication and improve the water quality of shallow lakes [18,22,23], and a variety of ecological restoration technologies have been developed and widely applied to shallow lakes, including biological preparation methods, biological methods, and ecological engineering restoration methods [24,25]. Different methods provide different principle characteristics. Biological preparation methods, such as effective microorganisms [26], improve the water quality to control cyanobacteria by using inhibitory algal agents or microorganisms. Biological methods, such as the protection and restoration of submerged macrophytes that compete with phytoplankton for nutrients [18,27,28], make use of biological interactions and competition between organisms to sustain the clear water state. The ecological engineering restoration method has drawn special attention, which combines hydraulics with ecology to ensure ecosystem sustainability and to return an ecosystem to a relatively natural state. This method is a new and innovative repair method that includes practices such as artificial wetlands of coastal belts and biological floating island technology [29,30]. Given the variety of available technologies, however, our lack of a reliable quantitative understanding of the consequences of different management alternatives often causes great difficulty in identifying effective measures to control the eutrophication of specific lakes [31]. Although these measures have received good feedback in some lakes of China, recent evidence from a few lake observations has demonstrated that a high degree of uncertainty regarding their long-term effectiveness also exists, which leads the lack of control of the removal effects over a long time [31,32]. Additionally, in previous practices over long periods, the ecological engineering restoration method mainly considered the biological removal effects but ignored the effects of lake flow patterns or did not pay enough attention to the hydrodynamic conditions in shallow lakes affected by hydraulic structures considering ecological operation, which have a significant influence on pollutant behavior [3]. For instance, *Eichhornia crassipes* ecological engineering treatment was applied to Lake Dianchi to improve the water quality and mitigate eutrophication by reducing the concentrations of P and N [33]. However, concurrently, the freshwater was also polluted, and cyanobacteria were enriched in the eastern part of Lake Dianchi Caohai and the northern part of Lake Dianchi Waihai under the effects of adverse water flow conditions in August [34]. To overcome this problem, the utilization of advantageous hydrodynamic conditions may be a valuable strategy to improve the water environment because such conditions can improve the water replacement efficiency, decrease chlorophyll a (Chla), which is an indicator of phytoplankton biomass and pollutants, and even change essential hydraulic conditions, which are crucial in accelerating eutrophication. Accordingly, it is urgent to study the responses of hydraulic mechanisms to pollutants and utilize necessary hydraulic structures during scientific ecological operations to change adverse water flow conditions to restore polluted lakes.

Since China’s economic reform and its integration with the world economy in the early 1980s, the country experienced a dramatic change regarding its social development and economy. Moreover, the natural environment in China suffered from accelerated lake eutrophication, and the eutrophication in Lake Dianchi is considered the most serious [15,35]. By the end of the 1990s, more than 70% of the lakes were considered eutrophic, and this phenomenon has become even more severe in recent years [36,37]. Lake Dianchi consists of Lake Dianchi Caohai and Waihai, which are located in the north and south of Dianchi Lake, respectively. Although 29 tributaries flow into Lake Dianchi, there are only two water exports, one in Lake Caohai and one in Lake Waihai. Lake Dianchi has experienced a complete conversion from oligotrophic to eutrophic with poor water quality [35], and cyanobacterial accumulation has become serious in the eastern part of Lake Caohai and the northern part of Waihai in recent years. The response of cyanobacteria accumulation in Northern Waihai was analyzed by Ma [34], and their results revealed that the key impact of the accumulation of cyanobacteria was adverse water flow conditions. Some countermeasures have been proposed to slow cyanobacteria accumulation in Northern Waihai, while there is no conclusion on the formation mechanism of cyanobacteria accumulation and no research on the changes to flow patterns with the poor water quality in Lake Dianchi Caohai. Although Lake Dianchi Caohai accounts for approximately 3% of the water surface area of Lake Dianchi, it accepts 45% of the wastewater that flows into Lake Dianchi [38,39,40], and the water quality in Lake Dianchi Caohai is much worse than that in Waihai in terms of pollutant concentrations due to its small area and downwind location [35]. In addition, the water quality in the lake will also directly affect the water quality in the Pudu River, which is a large branch of the Jinsha River. Thus, it is of great significance to study the flow patterns in Lake Dianchi Caohai to better understand the mechanism of cyanobacteria accumulation and pollutant behaviors. Currently, there are few reports on this in China. Studies of Lake Dianchi Caohai are extremely limited. Wang [39] found a significant positive effect on the removal of N by *Eichhornia crassipes*, which indicated that the large-scale utilization of *E. crassipes* was practical for the removal of nutrients in Lake Dianchi Caohai. Zhou [41] also used 10-year monitoring data of Lake Dianchi Caohai from 2002 to 2011 to reveal that the nutrients were the major factor that influenced the fluctuation of water quality, and total phosphorus (TP) and total nitrogen (TN) were the specific pollutants of the lake. Moreover, the influences of meteorological conditions and water quality factors on Chla concentration that indicates phytoplankton biomass in Lake Dianchi Caohai were also analyzed by Wei [8], and their results showed that TP might be the most important factor for the control of phytoplankton biomass. Despite their importance, these previous studies, which focused on only water quality trends and bioremoval efficiency, neglected water flow-field dynamics. Therefore, these studies likely overlooked important aspects of how the hydrodynamics in Lake Dianchi Caohai influenced the removal of P, N, and cyanobacterial biomass, and many aspects of the hydrodynamics are still not adequately understood. Lake managers and the public have been puzzled by the lack of a significant water quality response to the flow pattern. Hence, studies on hydrodynamics and water quality modeling are of great value for providing quantitative support for future lake managers by linking management measures to hydraulic mechanisms and pollutant behavior responses in Lake Dianchi Caohai.

It is necessary to develop a better understanding of the hydraulic mechanisms that drive the pollutant behaviors in Lake Dianchi Caohai to effectively explore methods to reduce the risk of eutrophication and poor water quality. In this study, a depth-averaged two-dimensional hydrodynamic and water quality model (MIKE 21) was used to simulate the lake flow patterns. We selected TP and TN, in accordance with the direct circulation proposed by Wei [8] and Zhou [41], to predict the spatial distributions of NH_3_-N, TP, TN, and Chla by both considering and ignoring hydraulic structures (water replacement channel). The hydrodynamic processes and distribution of Chla were connected to analyze the hydraulic mechanisms driving the accumulation of cyanobacteria in Lake Dianchi Caohai. Moreover, different ecological operation scenarios were simulated to analyze the effects of water replacement channels on water quality improvement based on the opening and closing of different gates. We also discuss the potential risk of eutrophication in the central part of the lake, and suggestions for further improvements and the achievement of water quality targets are discussed. Based on the results from this study, optimal ecological operations and suggestions for lake restoration are proposed.

## 2. Materials and Methods

### 2.1. Study Area

**Figure 1:**
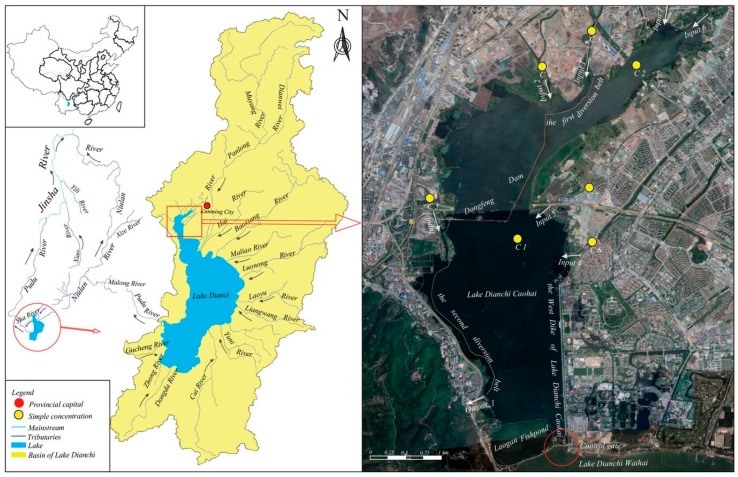
Location of the study area and sampling sites in Lake Dianchi Caohai (the red cycle means the enlarged area, and we received the maps from the Power China Kunming Engineering Corporation Limited and Google Earth).

Lake Dianchi, located in Southwest China (Yunnan Province), is a tectonic lake situated in the central part of the Yunnan-Guizhou Plateau and the sixth largest freshwater lake in China. Lake Dianchi Caohai, ranging from 24°57′ to 25°01′ N and from 102°37′ to 102°40′ E, is located in the northern part of Lake Dianchi and surrounded by the urban area of Kunming City, which is the capital of Yunnan Province, with a drainage area of 195 km^2^ (Figure 1). Lake Dianchi Caohai has a mean depth of 2.5 m, a surface area of 11.2 km^2^, and a storage capacity of 0.25 × 10^8^ m^3^, with a maximum length of 7.4 km (N-S) and a width of 2.3 km (W-E) at a normal water level of 1886.80 m above sea level. The lake is a typical wide and shallow plateau freshwater lake [14]. Moreover, the rivers that constantly supply water to Lake Dianchi Caohai include the Xinyunliang River (Input 1), Laoyunliang River (Input 2), Wangjiadui River (Input 3), Xiba River (Input 4), Chuanfang River (Input 5), Daguan River (Input 6), and Wulong River (Input 7), whereas there is only one outlet of the lake located in Xiyuan Tunnel (Output 1), which is located in southwestern Lake Dianchi Caohai. The length of the lake shoreline is approximately 23 km. The West Dike of Lake Caohai is formed by an artificial filling lake located on the eastern coast of the lake, which is an important landscape along the lake for the ecological and tourism value, with a length of 2.4 km (Figure 1). The mean annual precipitation is approximately 900–1100 mm, and more than half of that occurs from June to October.

In the 1960s, large-scale dam construction in Lake Dianchi resulted in the relative separation of Dongfeng Dam, Laogan Fishpond, and Caohai, and the West Dike of Lake Caohai was constructed [42,43]. The lakeside ecosystem in this area had been severely damaged due to the reclamation of land from the lake influences. By the 1980s, Lake Dianchi Caohai accepted most of the domestic sewage from Kunming City and the industrial wastewater from the western and southern suburbs through its tributaries during the rapid socioeconomic development of Kunming City. These feed-in tributaries were seriously polluted, resulting in the drastic deterioration of water quality in the lake. The water quality of the lake in the 1960s and 1970s met the Grade II and III requirements of the national standards (GB 3838-2002), respectively. In the late 1970s, the water quality further deteriorated; it decreased by approximately one grade in ten years, which indicated a sharp deterioration in the Lake Dianchi Caohai region. Furthermore, the water quality of Lake Dianchi Caohai had seriously degraded to worse than Grade V since the 1990s due to a combination of natural factors and human activities [40,44,45]. During the economic boom around Period 20 of the “Eleventh Five-Year Plan” (2005–2010), Lake Dianchi Caohai was the most polluted area in the Lake Dianchi region [39,40,46,47], and it was in a very severe state of eutrophication, which caused cyanobacterial blooms [39,48]. Consequently, the Kunming City government published several policies and regulations to restore and protect the ecological ecosystem of Lake Dianchi Caohai [44], i.e., the city’s ecosystems. To avoid urban flooding, the lake water level is regulated within a range from 1885.51 m to 1887.33 m above sea level; the water quality of the total water area of the lake needs to meet the Grade IV requirement of the national standards (GB 3838-2002) in 2020 [49,50]. To reach the target of returning the pond back to a lake, ecological wetlands were constructed, and *Eichhornia crassipes* ecological engineering projects [33,39] were applied to the lake to improve the water quality by removing N and P. Severe engineering and non-engineering measures (hereafter referred to as the six major projects) were applied to Lake Dianchi Caohai for ecological restoration, including sewage interception engineering, inflowing river renovation, agricultural nonpoint source management, ecological engineering restoration of the lake, lakebed sludge dredging ecological engineering, and interbasin water diversion projects [40]. For example, the Niulanjiang Water Diversion Project was implemented in 2015 [51], and the first and second diversion belts were constructed in 2016 (Figure 1), with lengths of 1.07 and 3.00 km, respectively, to replace the water and feed-in tributaries of the west bank of Lake Dianchi Caohai into the Xiyuan Tunnel outlet to implement sewage diversion. Despite the large amount of work undertaken, no significant reduction in lake eutrophication rates has been achieved [31]. Cyanobacterial blooms in the West Dike of Lake Caohai almost appear and are significant in August [40,52]; thus, the water replacement channel consists of 24 gates, as the ecological restoration project was proposed by the Yunnan Provincial government in 2017 to increase the water replacement rate. However, the effect of water replacement channels on lake flow patterns and water quality improvement is still unclear. Therefore, decision makers desperately need a scientifically sound way to quantitatively evaluate the response of lake water quality to ecological restoration to propose scientifically sound management measures for ecological operation. In this study, August was used as the simulation period to analyze the water quality improvements of the water replacement channel and the hydraulic driving mechanisms for the formation of cyanobacteria accumulation regions [40,52,53].

### 2.2. Depth-Averaged Two-Dimensional Numerical Model and Its Governing Equations

The depth-averaged two-dimensional MIKE 21 numerical model, which was developed by the Danish Hydraulic Institute [54], has been widely used to simulate water flow and convective contaminant transport in lakes, reservoirs, and rivers [1,55,56]. For example, the hydrodynamic and water quality model was applied to Baiyangdian Lake in China. Suggested simultaneous water transfers that accelerate water movement and water quality improvement by model analysis were proposed [1]. In addition, the suggested water transfer scheme is being more effectively implemented. The vertical acceleration, which can be ignored in the typical wide and shallow Lake Dianchi Caohai, makes it necessary to model the water flow and pollutant behavior in two dimensions [40,44], and the MIKE 21 model is best suited for two-dimensional free-surface flows, where vertical stratification in the water column can be neglected [54]. Therefore, a depth-averaged two-dimensional model of the area was established using the hydrodynamic module of MIKE 21 coupled with the transport module to simulate the hydrodynamics and concentration fields of Lake Dianchi Caohai.

#### 2.2.1. The Hydrodynamic Model

The hydrodynamic model is based on the solution of the two-dimensional incompressible Reynolds-averaged Navier–Stokes equations, which are subject to the assumptions of hydrostatic pressure, and the solution of the Boussinesq equation. The continuity equation and two horizontal momentum equations for the x and y components are written as follows:

(1) $$\frac{\partial h}{\partial t}+\frac{\partial h\bar{u}}{\partial x}+\frac{\partial h\bar{v}}{\partial y}=hS$$

(2) $$\begin{array}{r} \frac{\partial h\bar{u}}{\partial t}+\frac{\partial h{\bar{u}}^{2}}{\partial x}+\frac{\partial h\bar{v}\bar{u}}{\partial y}=f\bar{v}h-gh\frac{\partial \eta }{\partial x}-\frac{h}{\rho_{0}}\frac{\partial p_{a}}{\partial x}-\frac{gh^{2}}{2\rho_{0}}\frac{\partial \rho }{\partial x}+\frac{\tau_{sx}}{\rho_{0}}-\frac{\tau_{bx}}{\rho_{0}}-\\ \frac{1}{\rho_{0}}\left(\frac{\partial s_{xx}}{\partial x}+\frac{\partial s_{xy}}{\partial y}\right)+\frac{\partial }{\partial x}\left(hT_{xx}\right)+\frac{\partial }{\partial y}\left(hT_{xy}\right)+hu_{s}S \end{array}$$

(3) $$\begin{array}{r} \frac{\partial h\bar{v}}{\partial t}+\frac{\partial h\bar{u}\bar{v}}{\partial x}+\frac{\partial h{\bar{v}}^{2}}{\partial y}=-f\bar{u}h-gh\frac{\partial \eta }{\partial y}-\frac{h}{\rho_{0}}\frac{\partial p_{a}}{\partial y}-\frac{gh^{2}}{2\rho_{0}}\frac{\partial \rho }{\partial y}+\frac{\tau_{sy}}{\rho_{0}}-\frac{\tau_{by}}{\rho_{0}}-\\ \frac{1}{\rho_{0}}\left(\frac{\partial s_{yx}}{\partial x}+\frac{\partial s_{yy}}{\partial y}\right)+\frac{\partial }{\partial x}\left(hT_{yx}\right)+\frac{\partial }{\partial y}\left(hT_{yy}\right)+hv_{s}S \end{array}$$

(4) $$h\bar{u}=\int_{-d}^{\eta }udzh\bar{v}=\int_{-d}^{\eta }vdz$$

where t is the time (s); x, y are the Cartesian coordinates (m); η is the surface elevation (m); d is the still water depth; h is the total water depth (m), expressed as h=η+d; u and v are the flow velocity components in the x and y directions (m/s), respectively; $\bar{u}$ and $\bar{v}$ are the depth-averaged velocities in the x and y directions (m/s), respectively; f is the Coriolis parameter; g is the gravitational acceleration, which is equal to 9.81 m^2^/s; ρ is the density of water (kg/m^3^); $s_{xx}$, $s_{xy}$, $s_{yx}$, and $s_{yy}$ are the components of the radiation stress tensor; $p_{a}$ is the atmospheric pressure (N/m^2^); $\rho_{0}$ is the reference density of water (kg/m^3^); S is the magnitude of the discharge due to point sources; $\left(u_{s},v_{s}\right)$ is the flow velocity at which the water is discharged into the ambient water (m/s); $T_{xx}$, $T_{xy}$, $T_{yx}$, and $T_{yy}$ are components of lateral stresses, including viscous friction, turbulent friction, and differential advection; $\left(\tau_{bx},\tau_{by}\right)$ are the x and y components of bottom stresses (N/m^2^), respectively; $\left(\tau_{sx},\tau_{sy}\right)$ are the x and y components of surface wind stresses, respectively, which can be expressed as follows:

(5) $${\bar{\tau }}_{s}=\rho_{a}c_{d}\left|u_{w}\right|{\bar{u}}_{w}$$

where $\rho_{a}$ is the density of air; ${\bar{u}}_{w}=\left(u_{w},v_{w}\right)$ is the wind speed 10 m above the sea surface; $c_{d}$ is the drag coefficient, which was defined by Wu [57,58,59].

#### 2.2.2. The Water Quality Model

The depth-averaged two-dimensional advection-dispersion equation is written as

(6) $$\frac{\partial h\bar{C}}{\partial t}+\frac{\partial h\bar{u}\bar{C}}{\partial x}+\frac{\partial h\bar{v}\bar{C}}{\partial y}=hF_{C}-hk_{p}\bar{C}+hC_{s}S$$

where $\bar{C}$ is the concentration of the pollutant (mg/L); $k_{p}$ is the linear decay rate of the pollution (1/s); $C_{s}$ is the concentration of the pollutant at the source; $F_{C}$ is the horizontal diffusion term defined by

(7) $$F_{C}=\left[\frac{\partial }{\partial x}\left(D_{h}\frac{\partial \bar{C}}{\partial x}\right)+\frac{\partial }{\partial y}\left(D_{h}\frac{\partial \bar{C}}{\partial y}\right)\right]$$

where $D_{h}$ is the horizontal diffusion coefficient (m^2^/s), expressed as $D_{h}=A/\sigma_{T}$, where $\sigma_{T}$ is the constant Prandtl number. Here, $D_{h}$ was formulated by the scaled eddy viscosity formulation [54,60].

### 2.3. Data and Boundaries

**Figure 2:**
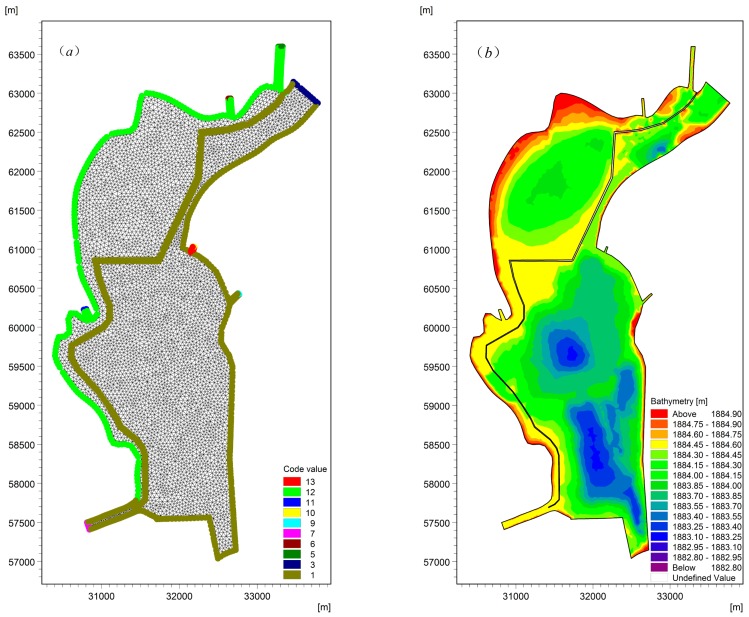
The computational mesh and bed elevation for Lake Dianchi Caohai ((**a**) is the computational mesh ignoring the water replacement channel, and (**b**) is the bed elevation ignoring the water replacement channel).

**Figure 3:**
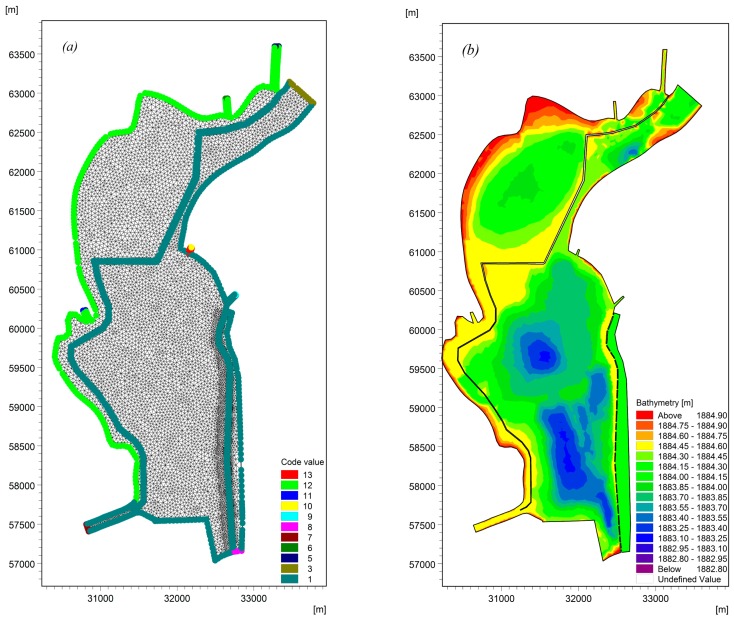
The computational mesh and bed elevation considering the water replacement channel for Lake Dianchi Caohai ((**a**) is the computational mesh considering the water replacement channel, and (**b**) is the bed elevation considering the water replacement channel).

To simulate the flow field and pollutant behavior of Lake Dianchi Caohai, monthly water quality data at seven water sampling sites from January 2016 to December 2016 were provided by the Central Monitoring Center of Kunming City (China) in this study (Figure 1). The seven water sampling sites include the Caohai Zhongxin (C1) site and Duanqiao (C2) site controlled by the Chinese government and five other sampling sites (C3–C7) representing the water quality of the tributaries flowing into the lake. The lake bed elevation data based on the Yellow Sea Datum were measured by the Power China Kunming Engineering Corporation Limited in 2016 for hydrodynamic and water quality models. The Laogan Fishpond, which is one part of the lake, is a closed region that is separated from dam construction. Furthermore, the water in this region is not exchanged with the lake water, and the water budget of the region is controlled by rainfall and evaporation. Therefore, the Laogan Fishpond was ignored in this study. In addition, to match the boundary conditions of the hydrodynamic model and water quality model, although the monthly water quality concentration data during the study periods were not available for the Daguan River and Wulong River, the Duanqiao (C2) site controlled by the Chinese government can represent the water quality after the confluence of the Daguan River and Wulong River. Consequently, the regions of the Daguan River and Wulong River were ignored during model validation, and only the main lake areas were considered. In this paper, unstructured grids were used to generate the lake mesh, with a minimum grid area of 0.038 km^2^. The lake mesh and terrain are shown in Figure 2 and Figure 3.

The water transferred from the Niulan River through the Daguan River and Xiba River is discharged into Lake Dianchi Caohai at Inputs 6 and 5, respectively (Figure 1). The daily input discharges provided by the Power China Kunming Engineering Corporation Limited at Input 1 to Inputs 5 and 8 (Duanqiao input) and monthly water quality concentrations at C2–C7 were used as the inflow boundaries. The lake outputs are located at the Xiyuan Tunnel (Output 1) and the water replacement channel (Output 2), as shown in Figure 3. The daily water level was used at the Xiyuan Tunnel (Output 1), and daily flow data were used at the water replacement channel (Output 2) in the hydrodynamic model; a zero gradient (Neumann boundary condition) was used as the outflow boundaries in the water quality model. The land boundary, including the lake shoreline and lake bed, was represented using zero normal velocity.

### 2.4. Initial Conditions

**Figure 4:**
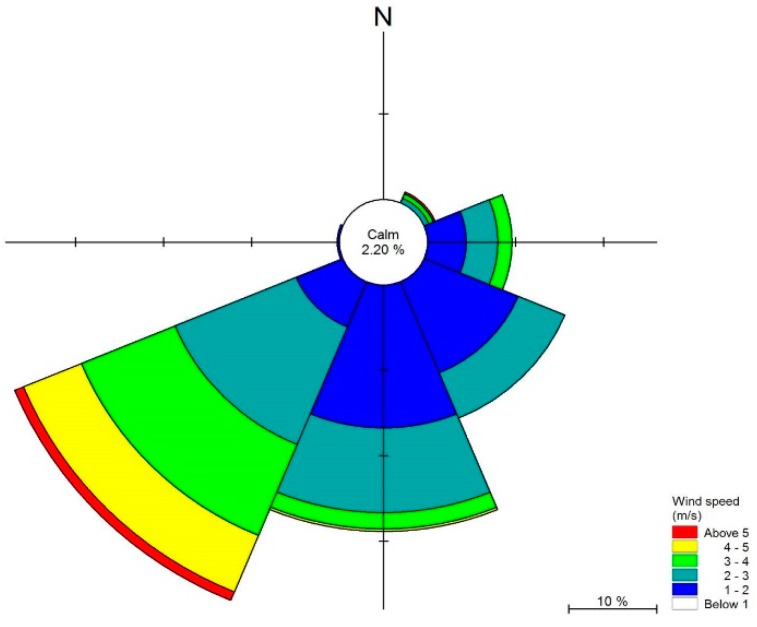
Wind rose map for Kunming City in 2016.

The flow patterns of Lake Dianchi Caohai are complicated due to the wind behavior, and wind has often been considered a predominant driving force, which affects the hydrodynamic conditions in shallow lakes [44,61,62]. Wind speed and wind direction data at one-day intervals for Kunming City were provided by the China Meteorological Data Service Center. The wind speed and wind direction in Kunming City at one-day intervals in 2016 are shown in Figure 4, which were used as the wind force to validate the hydrodynamic model and water quality model, indicating that the dominant wind direction is from the southwest (225°) and the dominant wind speed is 2–4 m/s. In particular, the average wind speed in the study area was 2.28 m/s, and the dominant wind direction of 225° was used in the models during the simulation period.

For the initial conditions, the water surface level is defined by the normal water level of the lake in the simulation period, the velocity component is defined as 0, and the initial concentrations of the water quality model are defined by the measured values. More parameters were determined via model validation.

### 2.5. Validation Procedure

In this study, the hydrodynamic model is validated using the relative error (R_e_) to evaluate the agreement between the model results and observations, and the water quality model is validated using the root mean square error (RMSE) and relative RMSE (RRMSE) [63]. The RMSE calculates the difference between the observed data $C_{i}$ and the value simulated by the water quality model $S_{i}$ for sample i. The RRMSE computes the ratio of the RMSE to the observed range of each water quality constituent and is expressed as a percentage [1,63]. The equations for the RMSE and the RRMSE are defined as follows:

(8) $$RMSE=\sqrt{\frac{1}{N}\sum_{i=1}^{N}{\left(C_{i}-S_{i}\right)}^{2}}$$

(9) $$RRMSE=\frac{RMSE}{O_{orange}}\times 100\%$$

where N is the total number of samples; $C_{i}$ is the observed water quality measurement; $S_{i}$ is the result simulated by the water quality model; $O_{orange}$ is the range of observed data computed from the maximum and minimum values.

Tang [1] employed an RRMSE less than 50% for nutrients to evaluate the simulation results of the MIKE 21 water quality model. Therefore, when evaluating the results obtained with the water quality model, an RRMSE less than 50% [1,63] was adopted to evaluate the comparison of the model simulated results and the observed measurements of the water quality constituent of the lake, which provides a straightforward statistic to evaluate the agreement between model results and observations.

## 3. Results and Discussion

### 3.1. Validation Results

The hydrodynamic model and water quality model were validated based on observation data from January 2016 to December 2016 via adjustment of the lake mesh and input parameters. The lake bed resistance was specified via a roughness Manning number (M) of 32 m^1/3^/s, which is the reciprocal value of the Manning coefficient n [64]. The horizontal eddy viscosity, with a value of 0.28, was set based on the Smagorinsky formulation for the whole lake domain [65]. The input values of the parameters for the hydrodynamic model are listed in Table 1.

The observed water level at the Caohai Zhongxin site where the operational water level was controlled by the “Regulations on the Protection of Dianchi in Yunnan” was chosen, and we compared the calculated values with the observed values. Furthermore, the accuracy of the hydrodynamic model was analyzed using the absolute error (A_e_) and the relative error (R_e_) of the water level at the site, which were less than 0.05 m and less than 0.1%, respectively. The results of this analysis reveal a close agreement between the model results and observations.

**Figure 5:**
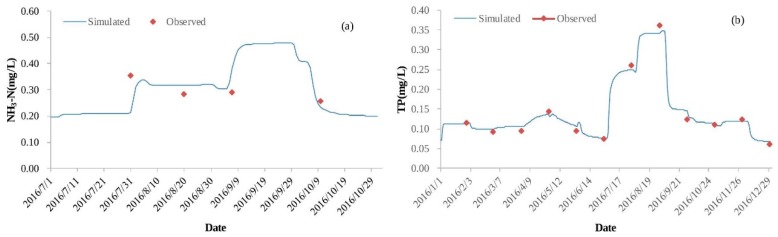

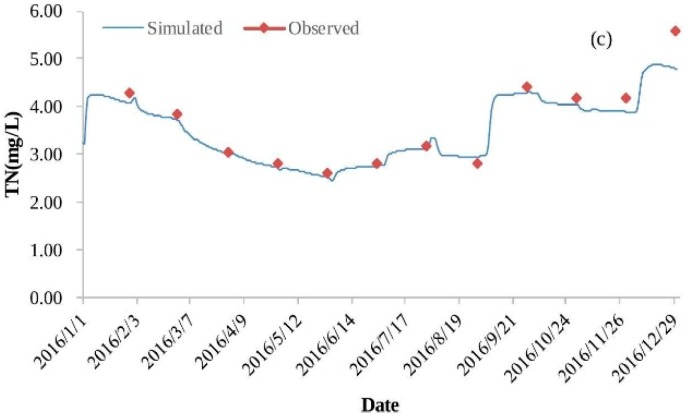
Validation of the water quality model at Caohai Zhongxin site ((**a**) is the validation of the NH_3_-N concentration, (**b**) is the validation of the TP concentration, and (**c**) is the validation of the TP concentration).

Figure 5 shows that the results of the simulation and observations exhibit the same changing trends for NH_3_-N, TP, and TN at the Caohai Zhongxin site. According to the validation of the water quality model, the scaling factor used to estimate the dispersion coefficient of all water quality constituents is set to 4, and the decay rates of NH_3_-N, TP, and TN are 0.007/d. The RMSE values of NH_3_-N, TP, and TN are 0.05 mg/L, 0.01 mg/L, and 0.26 mg/L, respectively. The RRMSE values of all water quality constituents are less than 50% at the Caohai Zhongxin site (Table 2), which indicates that the water quality model can simulate the pollution behavior in Lake Dianchi Caohai in this study, and this model can be used for further analysis.

**Figure 6:**
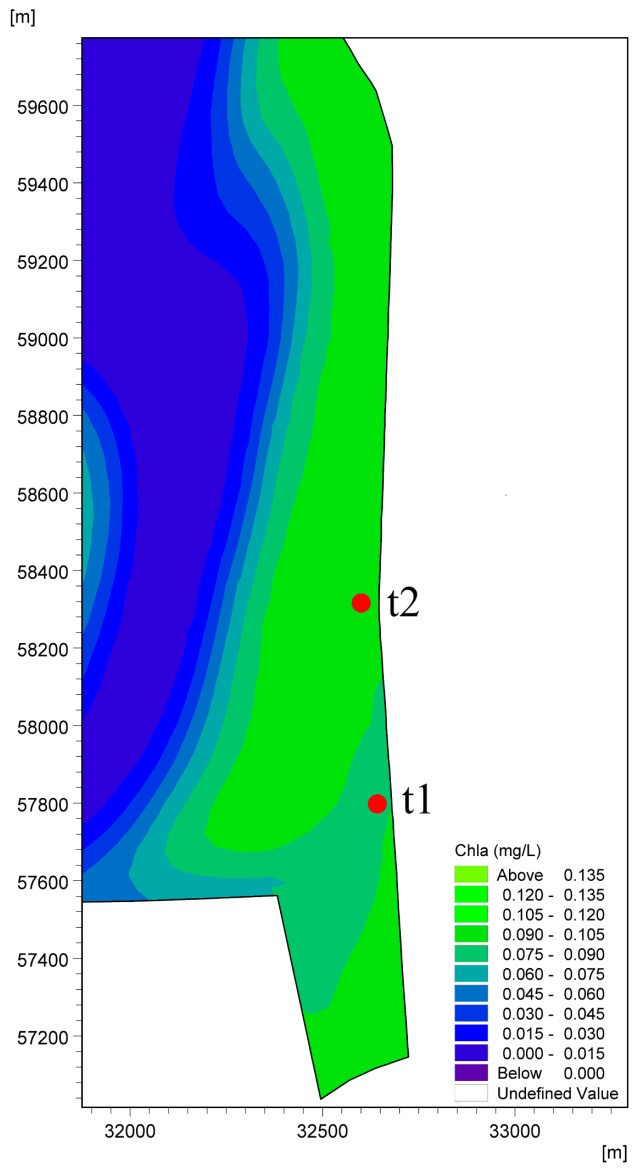
Validation of the distribution of Chla for the water quality model at Sites t1 and t2.

Table 3 also shows the distribution of Chla for validation of the lake water quality model. The observed concentrations of Chla at t1 (Jinjiadayuan site) and t2 (Hongta Western Road site) (Figure 6), which are both 50 m from the east shore and were provided by the Power China Kunming Engineering Corporation Limited in 2016, were chosen for comparison with the simulated results. The A_e_ values of t1 and t2 are −0.002 mg/L and 0.004 mg/L, respectively, and the R_e_ values are all less than 5.0%, which are in reasonably good agreement with the model results and observations.

### 3.2. Hydraulic Driving Mechanisms of the Accumulation of Cyanobacteria in Lake Dianchi Caohai

**Figure 7:**
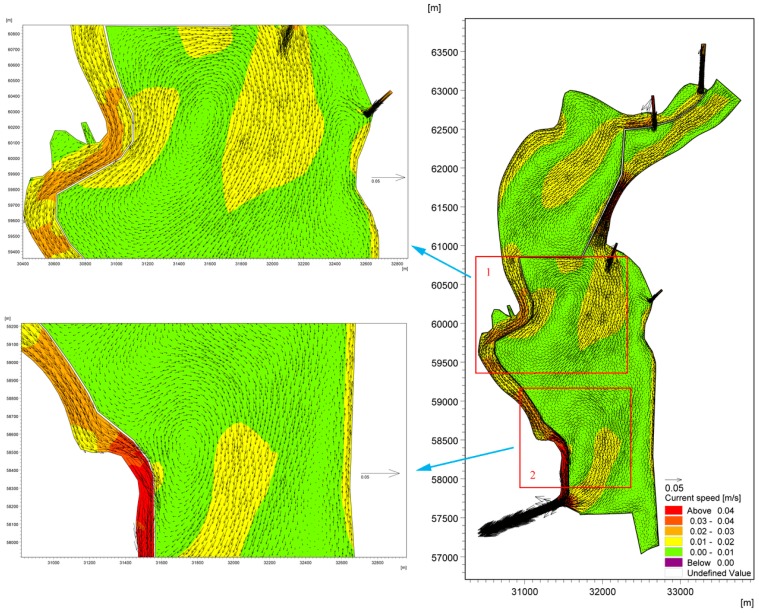
Lake flow field of Lake Dianchi Caohai considering wind force in August.

**Figure 8:**
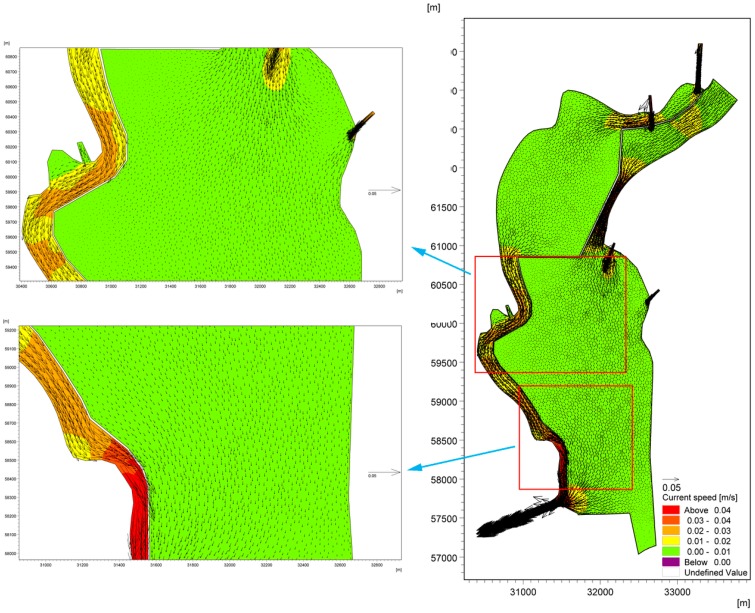
Lake flow field of Lake Dianchi Caohai ignoring wind force in August.

To adequately understand the hydraulic driving mechanisms and the primary factors that influence the accumulation of cyanobacteria in the West Dike of Lake Caohai, two models were developed to simulate the water flow fields that both consider and ignore wind force. One hydrodynamic model (Case A) was developed that considered both the tributary water input and wind force, and another model (Case B) used the same parameters as Case A but eliminated wind so that the wind stress effects were removed. The lake flow fields of Lake Dianchi Caohai considering and ignoring wind force in August are shown in Figure 7 and Figure 8, respectively. For the flow field without wind force, lake water flow is mainly driven by the inflow/outflow (water exchange with surrounding water bodies) [66], which is one of the primary forms of lake water movement [67,68], with water flows from the north to the south. The interval of the current speed in this lake is approximately 0.00–0.04 m/s, the maximum velocity occurs in the area between Dongfeng Dam and the eastern coast, and the velocity in the lake area, especially in the central part of the lake, is close to 0.00 m/s. To the south of Dongfeng Dam and the north of the control gate, the water is in a stationary state and cannot be replaced. Furthermore, no circulation occurs throughout the lake. We can speculate that the inflow/outflow without wind force can only slightly affect the flow pattern in the local water area near the entrances and exports at a regional scale (see Figure 8), as the water area tends to be narrower than the lake surface area over the entrances and exports. In contrast, for the flow field under the effects of the southwest wind force, there are two large-scale clockwise circulations (Circulations 1 and 2) in the central part of the lake (see Figure 7), with a current value of 0.01–0.02 m/s. The water along the West Dike of Lake Caohai flows from south to north under the influence of the dominant southwesterly wind, while in areas further away from the West Dike of Lake Caohai, the flow is in the opposite direction. There are many small counterclockwise circulations between the two main streams. The comparison of the flow field figures indicates that the only difference between the two model scenarios is whether there is spatially uniform wind stress; thus, we attribute the differences in the results between the cases solely to wind stress. It can be concluded that the lake water flow pattern is strongly controlled by wind stress, resulting in the formation of a distinctive mixed lake flow pattern in Lake Dianchi Caohai, which indicates that the flow pattern is dominated by wind-driven circulation and that the inflow/outflow plays only a subsidiary role during this period.

The formation of the wind-driven current of Lake Dianchi Caohai can be roughly divided into three stages: the stage of consistent lake water movement direction and wind direction, the stage of the formation of the rudiment of circulations, and the stable stage of circulation formation, which will affect the diffusion of pollutants. The first stage occurs in the early stage of wind action. Due to the drag effects of wind stress on lake water, the velocity of lake water gradually increases, consistent with direct wind forcing (SW). However, in some water areas with abrupt terrain changes near the shore, there are some different changes in the direction of water flow induced by the effects of the shore boundary, especially in the West Dike of Lake Caohai, where the lake water along the shore flows from south to north. Over time, the wind-driven current enters the second stage. The lake water is pushed to the downwind end by the persistent uniform wind force (SW); the gradual rise in the water level at the windward shore is attributed to the continuous accumulation of surging water at the downwind end, whereas the water level on the leeward side gradually decreases. If this northeastwardly water flow is sufficient in duration and direction, the water surface will tilt to balance the wind stress forcing, thereby creating a pressure gradient across the lake. This pressure gradient will result in the gradual deviation of the direction of water movement from the direction of the wind to seek equilibrium at the water surface and form the origins of the circulation. Subsequently, the wind-driven current enters the final stage. The water level and velocity field are constantly adjusted in response to the interaction among the pressure gradient force, Coriolis force, bottom stress, and wind stress and gradually reach dynamic equilibrium. In this state, the direction of the lake flow turns from deviating from the wind to gradually reversing. Moreover, steady-state circulations are formed, but in the West Dike of Lake Caohai, the depth of water along the shore is shallow, and the wind force is primarily responsible for the water flowing from south to north.

**Figure 9:**
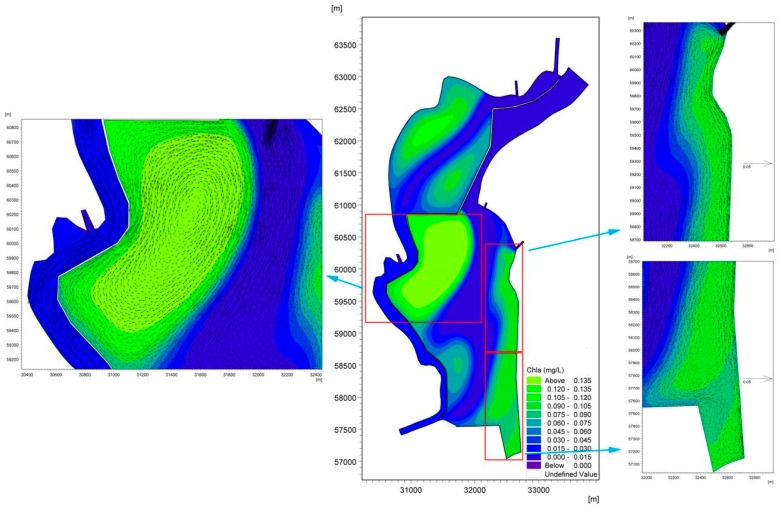
Accumulation diagram of Chla in the West Dike of Lake Caohai considering wind force in August.

**Figure 10:**
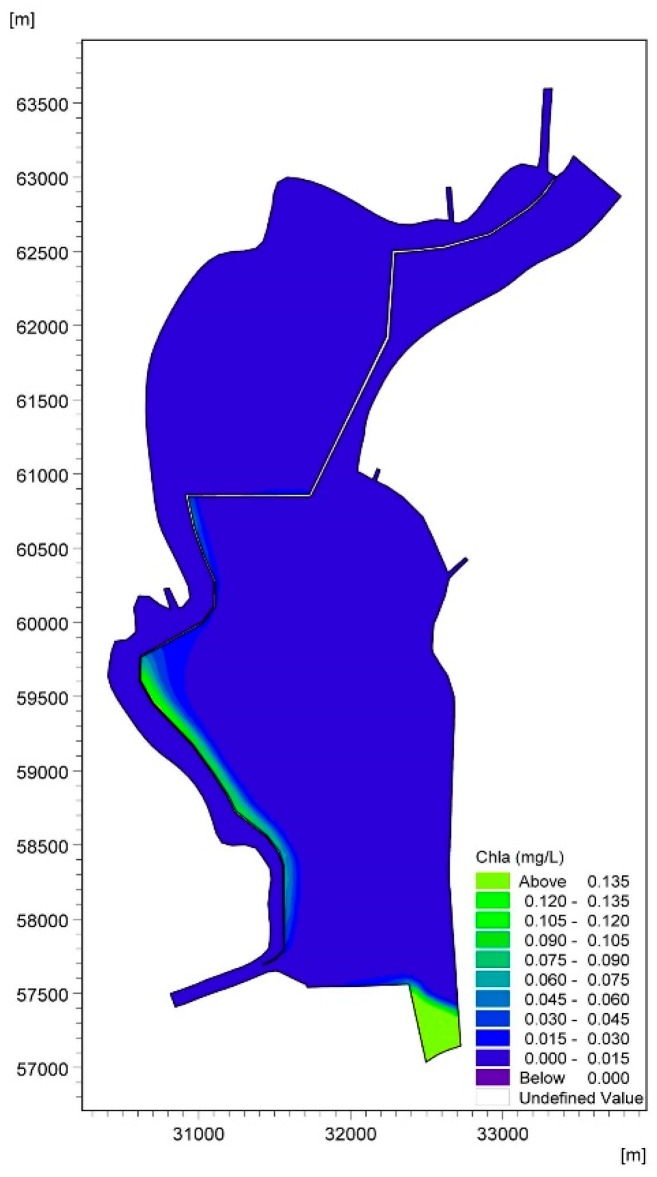
Accumulation diagram of Chla in the West Dike of Lake Caohai ignoring wind force in August.

Figure 9 shows the accumulation of cyanobacteria in the West Dike of Lake Caohai in August. It is not difficult to conclude that adverse hydrodynamic conditions are the main driving force for the formation of cyanobacteria accumulation areas in the West Dike of Lake Caohai. The cyanobacteria-enriched lake water constantly accumulates in the West Dike of Lake Caohai under the effects of strong southwest wind stress in the first stage of the formation of a wind-driven current. The cyanobacteria-enriched lake water flows from south to north in the West Dike of Lake Caohai when steady-state circulations are formed, which further enhances the accumulation of cyanobacteria. The cyanobacteria-enriched lake water that rests on the West Dike of Lake Caohai is induced by many small counterclockwise circulations resulting in the loss of hydrodynamic power that flows out of the lake with the mainstream (see Figure 9). The wind force response becomes important in determining the accumulation of cyanobacteria in the West Dike of Lake Caohai over long periods of time. Furthermore, there is almost no cyanobacteria-enriched lake water near the West Dike of Lake Caohai, except for the water near the control gate induced by the lentic conditions (see Figure 10). This result further shows that the accumulation of cyanobacteria in the West Dike of Lake Caohai is mainly due to wind effects, with an indication that inflow/outflow could play an active role in accelerating the replacement of cyanobacteria-enriched water.

Moreover, cyanobacteria-enriched water also accumulates to the south of Dongfeng Dam, resulting from the same initial concentrations throughout lake because observations of the Chla concentration are not available in this area of the lake. The cyanobacteria-enriched water cannot be replaced in the lake due to the effects of clockwise circulation 1 in the central part of the lake. We speculate that the accumulation of cyanobacteria will continue in this lake area if cyanobacteria blooms occur once in the south of Dongfeng Dam without any effective measures, which can remind lake managers to focus on scientific management in this region.

### 3.3. Water Quality Improvement Resulting from the Water Replacement Channel

The developed model was used to simulate the effects of the water replacement channel on water quality improvement in Lake Dianchi Caohai. To assure the best improvement in water quality, three ecological operation scenarios were evaluated in this study to discuss the optimal ecological operation after comparison with other possible operation scenarios: (1) opening of only the upper halves of the gates (hereafter referred to as UO); (2) opening of all the gates (hereafter referred to as AO); and (3) opening of only the lower halves of the gates (hereafter referred to as LO). The initial water level was set as 1886.80 m above sea level, and the initial water quality was set according to the observations. A wind speed of 2.28 m/s and a wind direction of 225° (SW) were set as the wind force in the simulation scenarios. According to the dispatching operation for Lake Dianchi Caohai issued by the Yunnan Provincial government, the discharge of the water replacement channel (Output 2) is 11 m^3^/s in August, while the other discharge was carried out via the Xiyuan Tunnel (Output 1) to meet the water level of 1886.80 m above sea level. Detailed parameters of the assumed scenarios are listed in Table 4. The assessment of the water environment of Lake Dianchi Caohai from 2015 to 2016 showed that the water quality of the lake degraded to worse than Grade V over long periods (GB 3838–2002). Hence, the input water quality of the lake tributaries was set based on observations in August to analyze the effects of water quality improvements when the water replacement channel was constructed in the three scenarios.

**Figure 11:**
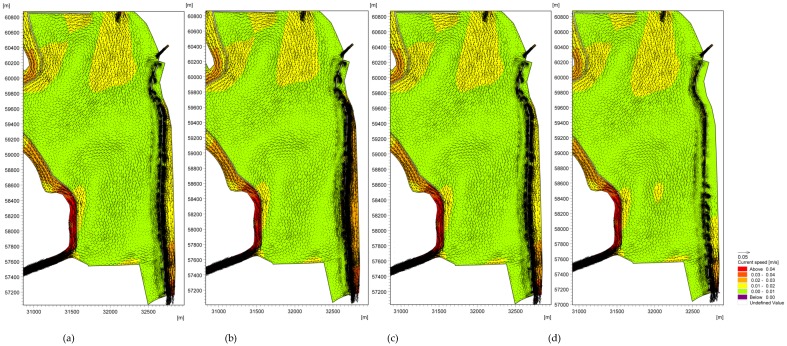
Lake flow fields of Lake Dianchi Caohai considering different ecological operations in August ((**a**) is the flow fields ignoring the water replacement channel, and (**b**) is the first scenario, (**c**) is the second scenario, and (**d**) is the third scenario. (**b**–**d**) consider the water replacement channel).

The hydrodynamic simulation results for the three ecological operation scenarios are shown in Figure 11. The water replacement channel accelerated the water changes. The modeling results indicate that two large clockwise circulations remain in the center of the lake, even when the water replacement channel is added as an export. We also found that the sizes and strengths of the circulations changed in the three scenarios. The circulation area was large and the velocity was reduced in three of the scenarios, resulting in impact regions that were larger than those in the scenario that ignored the water replacement channel (see Figure 11a); in particular, the circulation 2 moved eastward. Furthermore, along the West Dike of Lake Caohai, the small circulations mostly disappeared in all areas except for above the control gate when the flow velocity increased to 1.2–1.3 cm/s under the inflow/outflow effects due to the gates, which may result in negative effects to eutrophication in this region.

**Figure 12:**
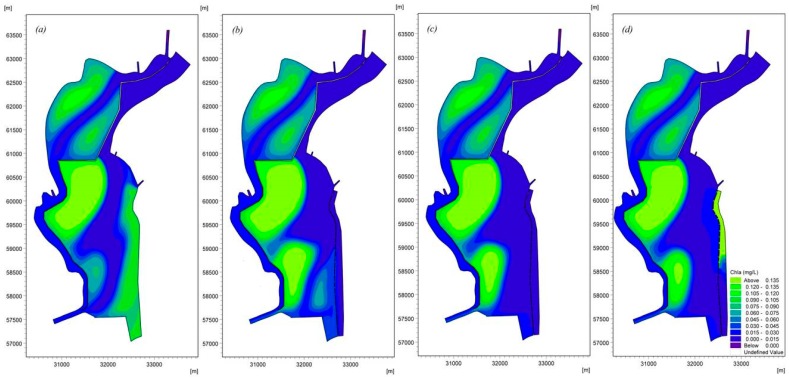
Spatial distribution of Chla considering different ecological operations in Lake Dianchi Caohai in August ((**a**) is the distribution of the Chla ignoring the water replacement channel, (**b**) is the first scenario, (**c**) is the second scenario, and (**d**) is the third scenario. (**b**–**d**) consider the water replacement channel).

**Figure 13:**
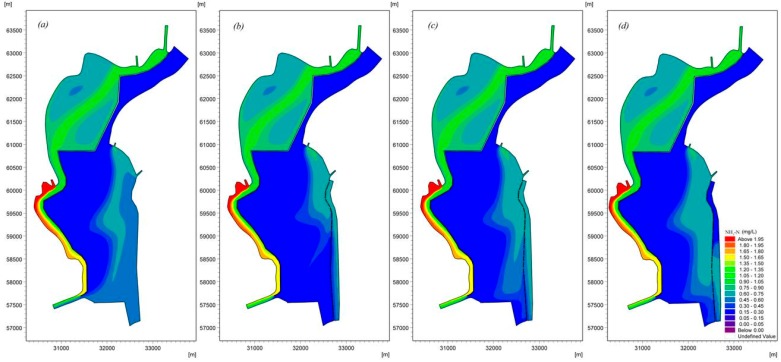
Spatial distribution of NH_3_-N considering different ecological operations in Lake Dianchi Caohai in August ((**a**) is the distribution of the NH_3_-N ignoring the water replacement channel, (**b**) is the first scenario, (**c**) is the second scenario, and (**d**) is the third scenario. (**b**–**d**) consider the water replacement channel).

**Figure 14:**
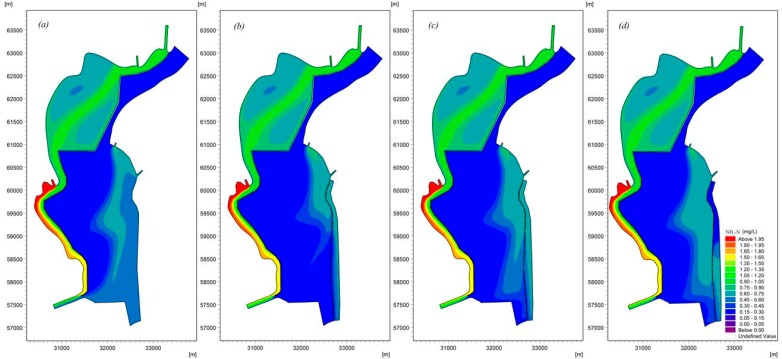
Spatial distribution of TP considering different ecological operations in Lake Dianchi Caohai in August ((**a**) is the distribution of TP ignoring the water replacement channel, (**b**) is the first scenario, (**c**) is the second scenario, and (**d**) is the third scenario. (**b**–**d**) consider the water replacement channel).

**Figure 15:**
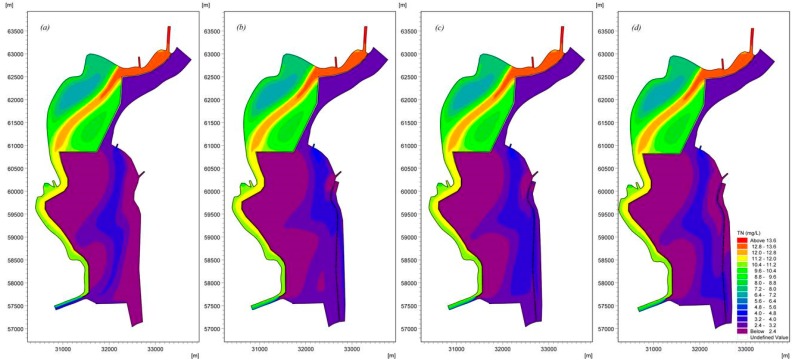
Spatial distribution of TN considering different ecological operations in Lake Dianchi Caohai in August ((**a**) is the distribution of TN ignoring the water replacement channel, (**b**) is the first scenario, (**c**) is the second scenario, and (**d**) is the third scenario. (**b**–**d**) consider the water replacement channel).

Figure 12, Figure 13, Figure 14 and Figure 15 demonstrate the water quality results based on the model under the three scenarios, which provide a basis for comparisons. Figure 12 shows the different effects of Chla removal in different scenarios. A better removal effect was obtained in each operation scenario, and the Chla removal effects of various operation scenarios were ranked as AO > LO > UP. The Chla concentrations along the eastern shore can meet the management target with a value of 0.035 mg/L [31] in the AO and LO scenarios, while the highest Chla concentration in the UP scenario is 0.06 mg/L, although the removal effect is good in this scenario. The improved removal effects are mainly due to the changes in the flow field induced by the enhancement of inflow/outflow along the eastern shore. We believe that the inflow/outflow destroys the small circulations, increasing the replacement rate of cyanobacteria-enriched water.

Cyanobacteria-enriched water accumulates not only to the south of Dongfeng Dam but also in the circulating at the bottom under the effects of the water replacement channel. The circulation 2 increases with the decrease in velocity due to the influence of inflow/outflow caused by the Xiyuan Tunnel, and this decrease leads to the decrease in the convection diffusion of Chla, resulting in Chla concentrations that cannot be reduced. These results suggest that the risk of cyanobacteria accumulation exists in this area. If cyanobacterial blooms occur in circulation 2 due to the effects of the water replacement channel, the Chla concentration cannot be reduced without effective measures under the effects of the water replacement channel. Thus, lake managers should pay additional attention to the formulation of appropriate management and risk response measures to ensure scientific management in this region.

The modeling results (Figure 13, Figure 14 and Figure 15) indicate that the water replacement channel will have a positive effect on water quality. Figure 13 presents the distribution of NH_3_-N in Lake Dianchi Caohai, illustrating that the removal of NH_3_-N differed considerably between the scenarios that considered and ignored the water replacement channel. The area with relatively poor water quality in the east decreased significantly. Moreover, the decrease in convection–diffusion in the region near the Xiyuan Tunnel that was due to decrease in the inflow/outflow at the Xiyuan Tunnel reduced the peak concentrations of NH_3_-N in the three scenarios by approximately 5.4%, 8.1%, and 6.8% and the minimum concentrations by approximately 0.0%, 5.0%, and 5.0%. Figure 14 suggests that the diversion/dilution that occurred due to the enhancement of inflow/outflow along the eastern shore reduced the peak TP concentrations in the three scenarios by approximately 20.5%, 23.1%, and 20.5% and the minimum concentrations by approximately 7.7%, 15.4%, and 0.0% throughout the lake. Figure 15 also shows the distribution of TN influenced by the water replacement channels under different ecological operations. The results demonstrate that the pollution zone moves eastward. Similarly, Figure 11 and Figure 15 show that lake flow fields are important in determining the TN concentration fields. This result can be attributed to the strength and size of the circulations resulting in the reduction in the peak TN concentrations in the three scenarios by approximately 1.8%, 6.1%, and 4.8% and the minimum concentrations by approximately 4.1%, 4.3%, and 4.1%. However, the influences of the different scheme combinations on the water area near the control gate are not very different. These results indicate that the water quality concentrations were generally much lower in the UO, AO, and LO scenarios than in the scenario that ignored the water replacement channel. The results of the current loading and input conditions in different scenarios demonstrate that the strength of the inflow/outflow effects along the eastern shore can directly affect the peak concentrations, especially for Chla and NH_3_-N, while the effects of inflow/outflow due to the Xiyuan Tunnel negatively impact the water quality. By comparing the results of all model scenarios, we believe that the AO scenario exhibits the best comprehensive effect for Chla, NH_3_-N, TP, and TN, and this scenario should be applied to Lake Dianchi Caohai for ecological operation during cyanobacteria outbreaks in August.

In addition, further comparison of concentration fields revealed that the water quality concentrations could not meet the management goals under the current loading and input conditions in all scenarios, regardless of the changes in the combination of ecological operation schemes. For a large and seriously polluted lake such as Lake Dianchi Caohai, changing the local flow field alone cannot improve the lake water quality to the desired status, which means that other measures such as water transfer engineering should also be considered, and load reduction will be fundamental for the improvement of lake water quality and ecological restoration. We will explore this topic in a follow-up study considering the diversion by hydraulic structures for further improvements to water quality to meet anticipated goals.

## 4. Conclusions

In this paper, a depth-averaged two-dimensional hydrodynamic and water quality model (MIKE 21) was developed using an unstructured grid to simulate the flow field and water quality of Lake Dianchi Caohai. The following conclusions were obtained through this research:

(1) Two large clockwise circulations were observed in the model results with many small counterclockwise circulations. These circulations show that the effects of wind force are primarily responsible for the mixing flow patterns in Lake Dianchi Caohai, resulting in the lake flow pattern being dominated by wind-driven circulation, while the inflow/outflow plays only a subsidiary role during this period. The formation of the wind-driven current in Lake Dianchi Caohai can be roughly divided into three stages. These results can be useful for quantitative water quality decision making.
(2) The validation results show that the hydrodynamic and water quality models can accurately simulate the hydrodynamic processes and pollution behaviors in Lake Dianchi Caohai. The modeling results show that wind is the predominant driving force that expedited water exchange and pollutant behavior, while water flow velocity was mainly driven by the inflow/outflow near the entrances and exports at a regional scale.
(3) By analyzing the hydrodynamic processes connected with the distribution of Chla, the adverse hydrodynamic conditions due mainly to wind effects are the predominant mechanism that determines the formation of cyanobacterial accumulation areas in the West Dike of Lake Caohai in August. Further analysis revealed that the accumulation of cyanobacteria would continue in this lake area if cyanobacteria blooms occur once in the south of Dongfeng Dam without any effective measures, and inflow/outflow would be advantageous for decreasing Chla.
(4) Based on the water quality model, a total of three ecological operation scenarios were simulated to distinguish the contributions of different operation scenarios to lake water quality. These scenarios were used to determine the optimal ecological operation for improving the water quality after comparing with other possible operation scenarios. The results show that the best comprehensive effect for Chla, NH3-N, TP, and TN occurs in the AO scenario, which should be applied for ecological operation in Lake Dianchi Caohai in August. Furthermore, this scenario can greatly reduce the Chla concentration, which indicates that cyanobacteria-enriched lake water can be completely replaced when all the gates are opened. These results provide a useful tool for quantitative water quality decision making.
(5) According to the modeling results, we suggest that the risk of cyanobacteria accumulation exists in the region south of Dongfeng Dam and in the circulation 2. Moreover, if cyanobacterial blooms occur in the lake region, the Chla concentration cannot be reduced without effective measures under the effects of water replacement channels; thus, lake managers should pay more attention to the formulation of appropriate management and risk response measures to scientifically manage this region.

This paper focuses on the flow patterns and responses of hydraulic mechanisms to pollutants, especially for the hydraulic driving mechanisms of cyanobacterial accumulation in Lake Dianchi Caohai. The research results play an important guiding role in studying the response of water quality to flow patterns, such as in Lake Dianchi Caohai. In addition, this restoration procedure in this paper can be applied to shallow lakes whose flow pattern was influenced by wind force and inflow/outflow. Furthermore, within a certain time period, the water replacement channel has good applicability for improving water quality in Lake Dianchi Caohai, especially for Chla and NH_3_-N along the eastern shore. In the future, it may be valuable to study the collective effects of a water replacement channel and water transfer engineering while considering ecological operations and to quantify the amount of eco-hydrological flow to further improve the water quality to meet anticipated goals.

## Figures and Tables

**Table 1:** Input values of the parameters for the MIKE 21 hydrodynamic model.

Parameters	Value
Minimum grid area of the mesh	0.038 km^2^
Manning number	32 m^1/3^/s
Horizontal eddy viscosity	0.28

**Table 2:** Quantitative assessment of model validation for the MIKE 21 water quality model.

Site		RMSE (mg/L)			RRMSE (%)	
	NH_3_-N	TP	TN	NH_3_-N	TP	TN
Caohai Zhongxin site	0.05	0.01	0.26	29.93	4.24	11.48

**Table 3:** Quantitative assessment of model validation for the distribution of Chla for the water quality model.

Sites	Chla (mg/L)			Error Analysis	
	Observed	Simulated		A_e_ (mg/L)	R_e_ (%)
t1	0.089	0.087		−0.002	−2.247
t2	0.089	0.093		0.004	4.494

**Table 4:** Scenario settings for ecological operation of Lake Dianchi Caohai.

Input Discharge (m^3^/s)		Input Water Quality (mg/L)				Output Boundaries			Initial Water Quality of Lake (mg/L)			
		NH_3_-N	TP	TN	Chla	(1)	(2)	(3)	NH_3_-N	TP	TN	Chla
Input 1	3.84	1.027	0.140	13.80	0	output 1: 1886.80 m above sea level	output 1: 1886.80 m above sea level	output 1: 1886.80 m above sea level	0.284	0.100	1.50	0.257
Input 2	3.33	1.027	0.140	13.80	0							
Input 3	0.63	10.18	0.800	11.20	0	output 2: opening of only the upper halves of the gates (11 m^3^/s)	output 2: opening of all of the gates (11 m^3/^s)	output 2: opening of only the lower halves of the gates (11 m^3^/s)				
Input 4	2.32	0.543	0.068	4.560	0							
Input 5	1.35	0.683	0.035	1.570	0							
Input 8	8.34	0.172	0.401	2.720	0							
Nonpoint	0.72	0.784	0.294	1.530	0							
